# Analysis of the effects of Cu-MOFs on fungal cell inactivation†

**DOI:** 10.1039/d0ra08743b

**Published:** 2021-01-04

**Authors:** Mayura Veerana, Hyun-Chul Kim, Sarmistha Mitra, Bishwa Chandra Adhikari, Gyungsoon Park, Seong Huh, Sung-Jin Kim, Youngmee Kim

**Affiliations:** Plasma Bioscience Research Center, Department of Electrical and Biological Physics, Kwangwoon University Seoul 01897 Republic of Korea gyungp@kw.ac.kr +82-2-940-5664 +82-2-940-8324; Department of Chemistry, Protein Research Centre for Bio-Industry, Hankuk University of Foreign Studies Yongin 17035 Republic of Korea; Institute of Nano-Bio Technology, Department of Chemistry and Nano Science, Ewha Womans University Seoul 03760 Republic of Korea ymeekim@ewha.ac.kr +82-2-3277-2385 +82-2-3277-4164

## Abstract

Three dimensional (3D) copper metal organic frameworks (Cu-MOFs) containing glutarates and bipyridyl ligands (bpa = 1,2-bis(4-pyridyl)ethane, bpe = 1,2-bis(4-pyridyl)ethylene, or bpp = 1,3-bis(4-pyridyl)propane) were synthesized by using previously reported hydrothermal reactions or a layering method. All three Cu-MOFs contained well-defined one dimensional (1D) channels with very similar pore shapes and different pore dimensions. The bulk purities of the Cu-MOFs were confirmed using powder X-ray diffraction (PXRD) and infrared spectroscopy (IR) spectra. When the three types of Cu-MOFs were applied to *Candida albicans* cells and *Aspergillus niger* spores, an average of about 50–80% inactivation was observed at the highest concentration of Cu-MOFs (2 mg mL^−1^). The efficiency of the fungal inactivation was not significantly different among the three different types (bpa, bpe, bpp). Treatment of the fungi using Cu-MOFs induced an apoptosis-like death and this was more severe in *A. niger* than *C. albicans*. This may be due to elevation of the intracellular level of reactive oxygen species (ROS) in *A. niger*. Generation of the reactive species in solution by Cu-MOFs was observed. However, there was a dramatic variation in the levels observed among the three types. Our results suggest that Cu-MOFs can produce antifungal effects and induce apoptosis-like death of the fungi, which was probably caused by the elevated level of intracellular reactive species.

## Abstract 2

Cu-MOFs containing glutarates and bipyridyl ligands produce antifungal effects on *C. albicans* cells and *A. niger* spores, and induce apoptosis-like death of the fungi, which was probably caused by the elevated level of intracellular reactive species.

## Graphical abstract

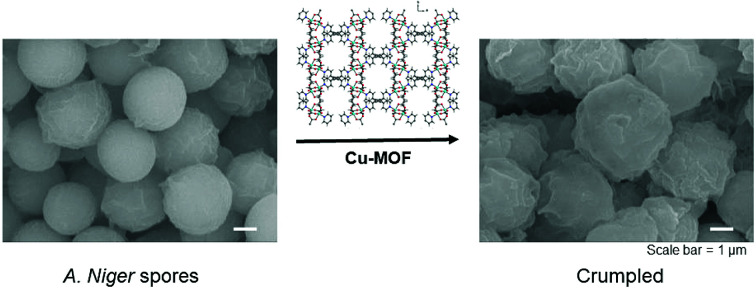

## 1. Introduction

Metal–organic frameworks (MOFs) and coordination polymers (CPs) have attracted considerable interest in coordination and materials chemistry,^1^ and well-known applications of MOFs include gas sorption and separation,^2^ catalysis,^3^ luminescence sensing,^4^ and biomedical usage.^5^ These MOFs can also be used as bioactive framework materials (BioMOFs)^5,6^ and they have received considerable attention for the development of novel and efficient antimicrobial agents. Silver coordination networks have been used as effective antimicrobial agents, and have shown notable antimicrobial efficiency against a range of different bacteria (*E. coli*, *P. aeruginosa* and *S. aureus*) and yeast (*C. albicans*) by releasing Ag^I^ ions from Ag-MOFs or Ag-CPs.^7^ The water-stable Cu-BTC (1,3,5-benzenetricarboxylate) metal organic framework (MOF) can inhibit the rate of growth of *C. albicans*,^8^ and another Cu-based HKUST-1 MOF can inactivate *Saccharomyces cerevisiae*, the antifungal property of which is a result of Cu^II^ ions that are released from its structure.^9^ A Co-based MOF (Co-TDM, TDM^8−^ = [(3,5-dicarboxyphenyl)-oxamethyl]methane), was shown to be highly effective at inactivating *E. coli*.^10^

Recently, Co^II^-coordination polymers (Co-CPs) containing glutarates and bipyridyl ligands (bpa = 1,2-bis(4-pyridyl)ethane, bpe = 1,2-bis(4-pyridyl)ethylene, or bpp = 1,3-bis(4-pyridyl)propane) have been prepared.^11^ Co-CPs can inactivate *C. albicans* cells more efficiently than *A. niger* spores within the same treatment time, and the greater inactivation of *C. albicans* by Co-CPs may result from dramatic changes in the morphology of *C. albicans*. We concluded that the small amount of leached Co^II^ ions and the robust Co-CPs themselves, as well as the reactive species generated by the Co-CPs, can actively participate in fungal inactivation.

**Scheme 1:**
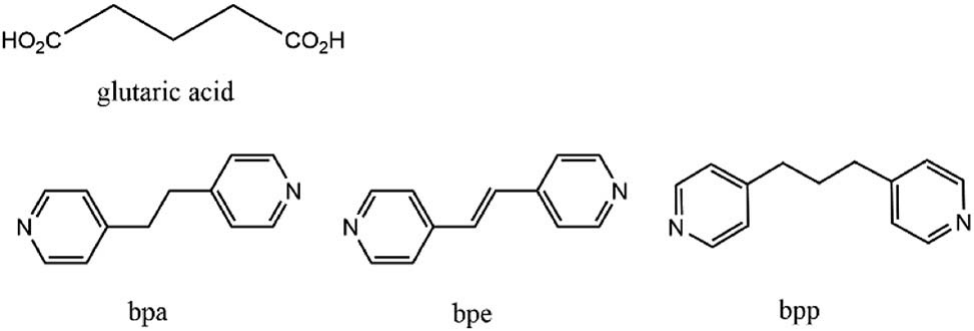
Chemical structures of glutaric acid, bpa, bpe, and bpp ligands.

As an extension of our previous work, we tested the antifungal properties of Cu-MOFs, with the same glutarate and bipyridyl ligand system (Scheme 1), against *C. albicans* and *A. niger*. In contrast to the Co-CPs, all three Cu-MOFs, with the formulation [Cu_2_(Glu)_2_(μ-L)]·x(H_2_O) (L = bpa, bpe, or bpp), have similar 3D frameworks with well-defined one dimensional (1D) channels and possess very similar pore shapes with different pore dimensions and void volumes.^12^ These Cu-MOFs showed excellent antibacterial activities against the five types of bacteria tested, including both Gram-positive bacteria (*Staphylococcus aureus* and MRSA) and Gram-negative bacteria (*Escherichia coli*, *Klebsiella pneumoniae*, and *Pseudomonas aeruginosa*), with very low MBCs (minimal bactericidal concentrations).^12^ The antifungal properties of the Cu-MOFs can be comparable to those of the Co-CPs containing the same ligand system.

## 2. Experimental

### 2.1 Instrumentation

Powder X-ray diffraction (PXRD) patterns were recorded on a Bruker D8 Focus diffractometer (40 kV, 30 mA, step size = 0.02°). Infrared spectroscopy (IR) spectra were measured on a BIO RAD FTS 135 spectrometer using KBr pellets. Inductive coupled plasma mass spectrometer (ICP-MS) measurements were performed in the Seoul Center, Korea Basic Science Institute.

### 2.2 Fungal strains and culture conditions

The fungal strains used in the study were *C. albicans* KCTC7270 (yeast type) and *A. niger* which were kindly provided by the Korean Collection for Type Cultures in Korea Research Institute of Bioscience and Biotechnology (Jeongeup-si, Jeollabuk-do, Korea) and Dr. Seong-Hwan Kim's Laboratory in Dankook University (Cheonan-si, Chungnam, Korea), respectively. The fungi were grown and maintained in potato dextrose agar (PDA) at 30 °C.

### 2.3 Treatment with Cu-MOFs and assay for fungal viability

*C. albicans* cells and *A. niger* spores were treated with Cu-MOFs containing glutarates and bpa (MOF 1), bpe (MOF 2) or bpp (MOF 3). *C. albicans* cells were prepared as follows, 1–2 colonies from the PDA culture plate were suspended in 1 mL of potato dextrose broth (PDB), and 10 μL of the suspension was inoculated into a further 15 mL of PDB. After incubation at 30 °C with shaking for 20 h, the cells were pelleted down *via* centrifugation at 3134 × *g* for 5 min. The liquid part was discarded, and the cell pellet was washed with 1× PBS (phosphate buffered saline). Then, fresh PBS was added to the washed cell pellet and the concentration was adjusted to 10^8^ per mL. To collect the spores of *A. niger*, about 10 mL PBS was added into a PDA culture plate grown for 2 weeks, and the fungal mycelia were scrapped using scrapper. The scrapped suspension was filtered through three layers of miracloth. The filtrate was centrifuged at 3134 × *g* for 5 min and the liquid part was discarded. The spore pellet was washed with PBS and resuspended in new PBS to give a concentration of 10^8^ per mL.

*C. albicans* cells and *A. niger* spores suspended in PBS at a concentration of 10^8^ per mL were added to each well of a 24 well plate (1 mL per well). Stock suspensions of MOF 1, MOF 2 and MOF 3 were prepared by adding MOF powders in PBS (20 mg mL^−1^). After well mixed, an appropriate volume of MOF stock suspension was added into each well with final concentrations 0, 0.125, 0.25, 0.5, 1, and 2 (mg mL^−1^). The plates were incubated at room temperature with shaking for 4 d.

After treatment with Cu-MOFs, 10 μL of the fungal suspension was removed and serially diluted, and then 100 μL of the diluted suspension was spread onto the PDA plate. The plates were incubated at 30 °C for 2 d, and the number of colonies was counted. The number of colonies from nine replicate plates was averaged.

### 2.4 Scanning electron microscopy analysis

The surface morphology of the *C. albicans* cells and *A. niger* spores was analysed using scanning electron microscopy (SEM) after treatment with Cu-MOFs (2 mg mL^−1^) at room temperature with shaking for 4 d. The untreated sample was used as a control. The treated and control fungal cells and spores were prepared for SEM analysis by following previously described procedures.^10^ The prepared samples were examined under a scanning electron microscope (JEOL, Tokyo, Japan).

### 2.5 Measurement of the levels of reactive oxygen species and reactive nitrogen species

To measure the levels of the intracellular reactive species, *C. albicans* cells and *A. niger* spores in PBS (10^8^ mL^−1^) were treated with Cu-MOFs (2 mg mL^−1^) for 4 d as described in an earlier section. An untreated sample was used as a control. The treated and control cells and spores were washed with PBS once and incubated in PBS containing 10 mM of H_2_DCFDA or DAF-FM DA at room temperature in the dark for 1 h. After incubation, the fungal cells and spores were washed with PBS twice and resuspended in 100 μL of PBS. The fungal suspensions were transferred to a black walled 96-well assay plate, and the fluorescence was measured at 495 nm (excitation) and 515 nm (emission) using a Synergy HTX Multi-Mode Reader (BioTek Instruments, VT, USA).

To measure the levels of H_2_O_2_ and NO_*x*_ in the media, 1 mL of PBS was treated with the Cu-MOFs (2, 1, 0.5, 0.25, 0.125, and 0 mg mL^−1^) at room temperature with shaking. After treatment, the level of H_2_O_2_ and NO_*x*_ was measured immediately (0 d) and on the 4^th^ day. An Amplex™ Red Hydrogen Peroxide/Peroxidase Assay Kit (Molecular Probes, Eugene, OR, USA) and QuantiChrom™ Nitric Oxide Assay Kit (BioAssay Systems, Hayward, CA, USA) were used to determine the H_2_O_2_ and NO levels, respectively, following the manufacturer's protocols.

### 2.6 TUNEL assay

Apoptosis-like fungal cell death was analysed using the TUNEL (terminal deoxynucleotidyl transferase dUTP nick end labelling) assay. In the TUNEL assay, DNA fragmentation was detected by labelling the 3′-hydroxy terminals with fluorescence. *C. albicans* cells and *A. niger* spores in PBS (10^8^ mL^−1^) were treated with Cu-MOFs (2 mg mL^−1^) at room temperature with shaking for 4 d. An untreated sample was used as a control. The fungal cell wall was first removed before the TUNEL assay. The treated and control fungal cells and spores were washed with PBS and then incubated in OM buffer (1 M MgSO_4_, 8.4 mM Na_2_HPO_4_, 1.6 mM NaH_2_PO_4_) containing a lysing enzyme (10 mg mL^−1^, Sigma, St. Louis, MO, USA) at 30 °C for 2 h to digest the cell wall. After the cell wall had been digested the fungal protoplasts were washed with PBS once and fixed with 1% (v/v) paraformaldehyde in PBS on ice for 15 min. After fixation, the protoplasts were washed with PBS twice and resuspended in 0.1 mL PBS. 1 mL of ice-cold 70% (v/v) ethanol was added, this was incubated on ice for 30 min and then stored at −20 °C until further use.

The fungal protoplasts were further processed using the TUNEL Assay Kit - FITC (Abcam, Cambridge, UK). Ethanol was removed and the protoplasts were washed twice with wash buffer. After washing, 51 μL of the DNA labelling solution containing 10 μL of the reaction buffer, 0.75 μL of the TdT enzyme, 8 μL of FITC-dUTP, and 32.25 μL of ddH_2_O were added to the samples and then incubated at 37 °C for 3 h. After incubation, the protoplasts were washed twice with rinse buffer, resuspended in 0.1 mL of propidium iodide/RNase A and incubated at room temperature in the dark for 30 min. The fluorescence was observed *via* a confocal laser scanning microscope (Olympus Corporation, Tokyo, Japan) at 488 nm (excitation) and 520 nm (emission).

### 2.7 Quantitative PCR for the metacaspase gene

The transcription levels of the metacaspase-1 (ref. 13) in *C. albicans* and metacaspase 1A and 1B in *A. niger* (homologs of CasA and CasB in *A. fumigatus*, respectively)^14^ were assessed in the fungal samples treated with Cu-MOFs for 1 d using the quantitative polymerase chain reaction (QPCR). The total RNA extraction and QPCR were performed as described in a previous study.^15^ The actin gene was used as a reference. The sequences of the primer used in the QPCR were obtained as follows:

For *A. niger*:

Metacaspase-1A: forward-ATCCTCAAGGAGCCCAACTT, reverse-CTGGTCTTGGTCTGGATGGT

Metacaspase-1B: forward-CATCCAATTACCGCTTCCAG, reverse-CATCGGGTTCTTCTGATCGT

Actin: forward-ACAGTCCAAGCGTGGTATCC, reverse-GCCTGGATGGAGACGTAGAA;and for *C. albicans*:

Metacaspase-1: forward-CCACCAAGTCAACAAGGACA, reverse-GCCTTTTTCCGACCACTACA,

Actin: forward-AGGTTTGGAAGCTGCTGGTA, reverse-GAAGATGGAGCCAAAGCAGT.

## 3. Results and discussion

### 3.1 Structural properties of the Cu-MOFs containing glutarate and bipyridyl ligands

**Fig. 1:**
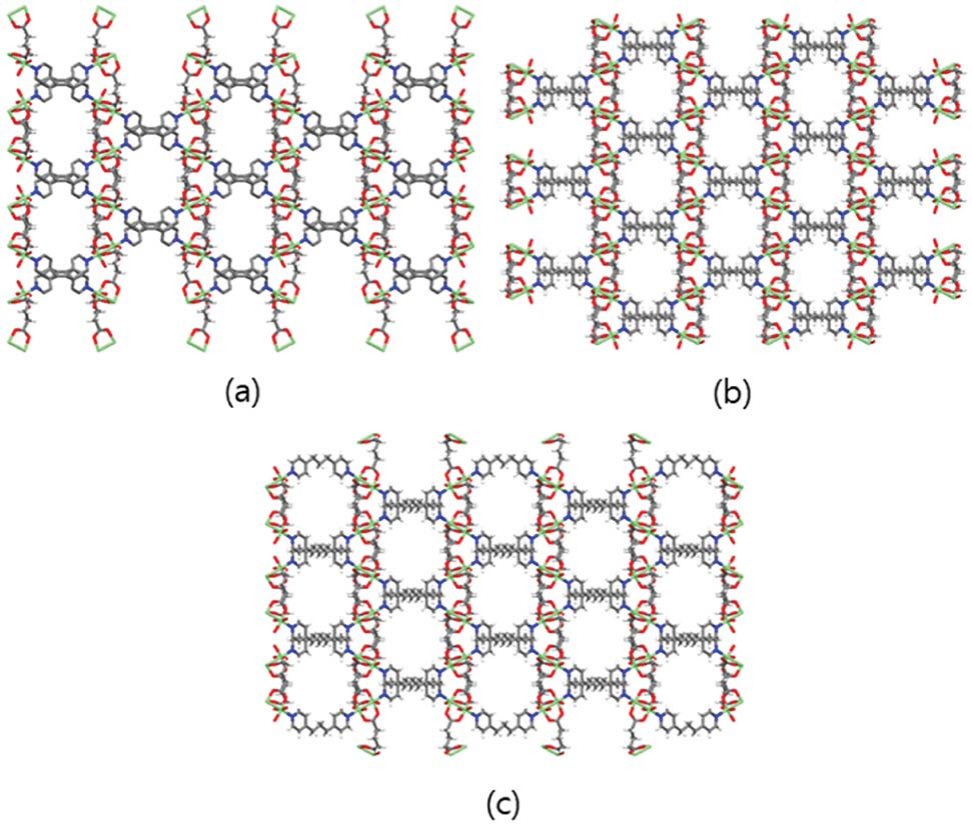
3D frameworks of MOF 1 (a), MOF 2 (b), and MOF 3 (c). Water solvent molecules have been omitted for clarity. Colour code: Cu: green; O: red; N: blue; C: grey; and H: white.

The 3D Cu-MOFs were synthesized by using previously reported hydrothermal reactions or a layering method.^12^ The paddle-wheel Cu_2_ dinuclear units in all three Cu-MOFs are connected by glutarates (Glu) to form 2D sheets, and these sheets are bridged by bipyridyl ligands (bpa, bpe, and bpp) to form the 3D frameworks (Fig. 1). The 3D frameworks had the formula [Cu_2_(Glu)_2_(μ-L)]·*x*(H_2_O), in which L is bpa (MOF 1), bpe (MOF 2) or bpp (MOF 3). All three MOFs contained well-defined 1D channels, and they possessed very similar pore shapes with different pore dimensions and void volumes.^12^

**Fig. 2:**
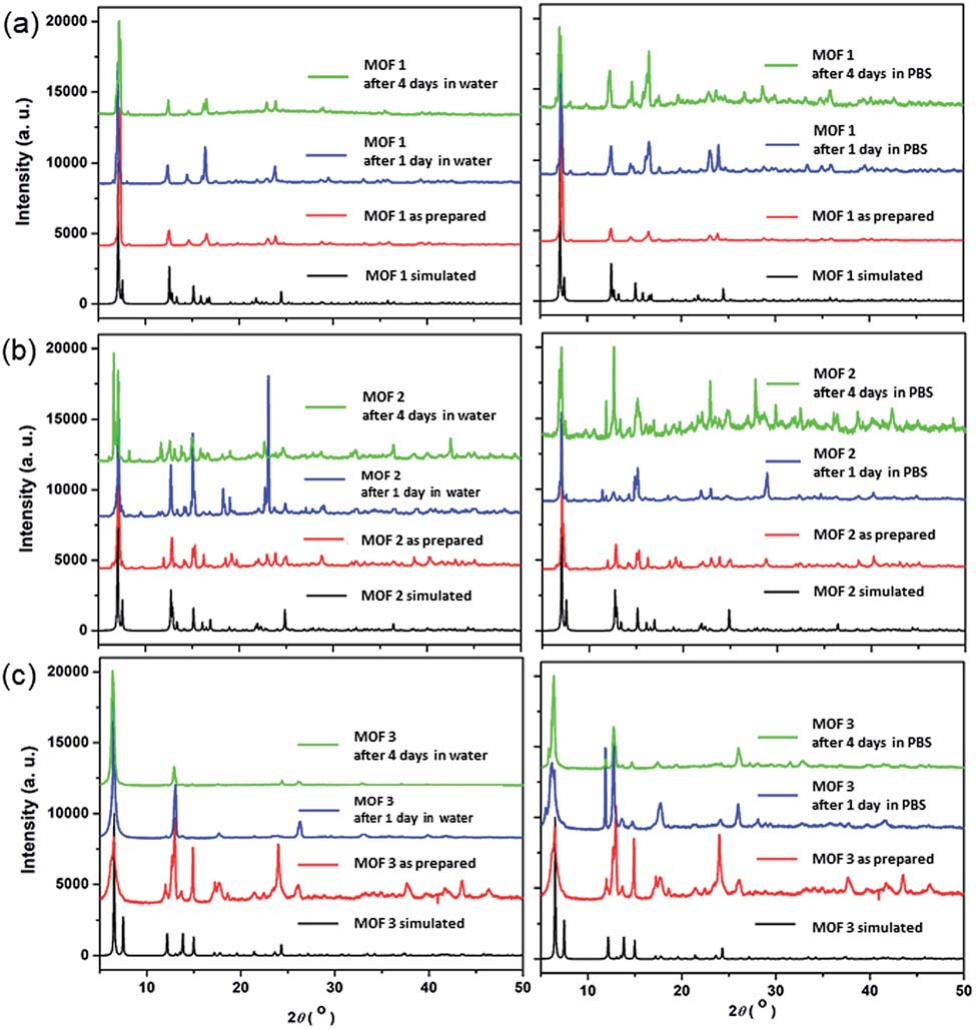
PXRD patterns of the Cu-MOFs: (a) MOF 1; (b) MOF 2; and (c) MOF 3; after 1 d and 4 d in water and 1× PBS solution.

The bulk purities of the Cu-MOFs 1–3 were confirmed using PXRD (Fig. 2), and the IR spectra of Cu-MOFs 1–3 were found to be in good agreement with those reported previously (Fig. S1†).^12^ Very strong, slightly broader bands were observed at approximately 1620 and 1420 cm^−1^, which represented the asymmetric and symmetric C=O stretching modes of the bound glutarate moieties. The absence of any bands in the area of approximately 1710 cm^−1^ indicated full deprotonation of all of the carboxylate groups in Cu-MOFs 1–3,^16^ which is consistent with the results of the X-ray analyses. The structural stabilities of MOFs 1–3 after 4 d in water or PBS solution were tested (Fig. 2). The PXRD patterns of MOFs 1–3 indicate that their structures are stable after 4 d in water or PBS solution.

**Fig. 3:**
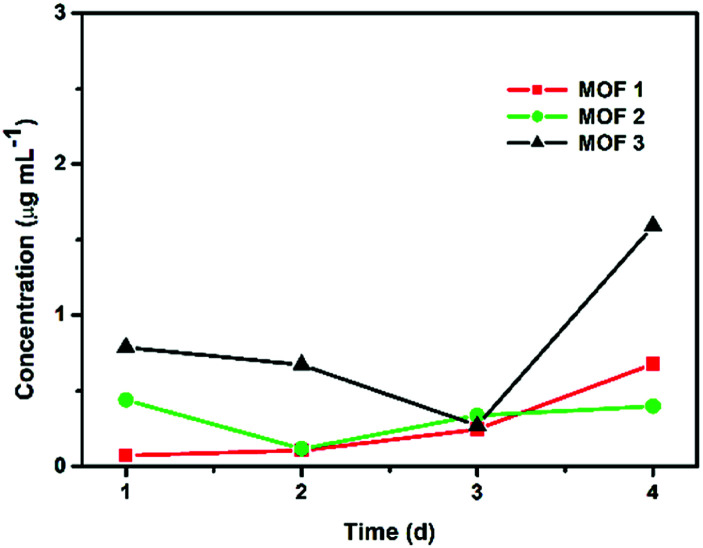
Concentrations of Cu^II^ ions released from 1 mg of Cu-MOFs in 1 mL of PBS.

The degradation tests were carried out in the PBS solution used for antifungal testing at room temperature to confirm that the leaching of Cu^II^ ions from Cu-MOFs was not significant. The quantities of Cu^II^ ions released from the Cu-MOFs were monitored using ICP-MS after 1, 2, 3 and 4 d (Fig. 3). The concentration of the released Cu^II^ ions from 1 mg of a compound in 1 mL of 1× PBS after 4 d was approximately 0.678 μg mL^−1^ for MOF 1. The amount of Cu^II^ ions released from MOF 1 (0.170 μg mL^−1^ = 0.00017 mg mL^−1^) is negligible compared to 2 mg mL^−1^. The Cu-MOFs do not easily release Cu^II^ ions and are stable in PBS.

### 3.2 Reduced fungal viability after treatment with Cu-MOFs

**Fig. 4:**
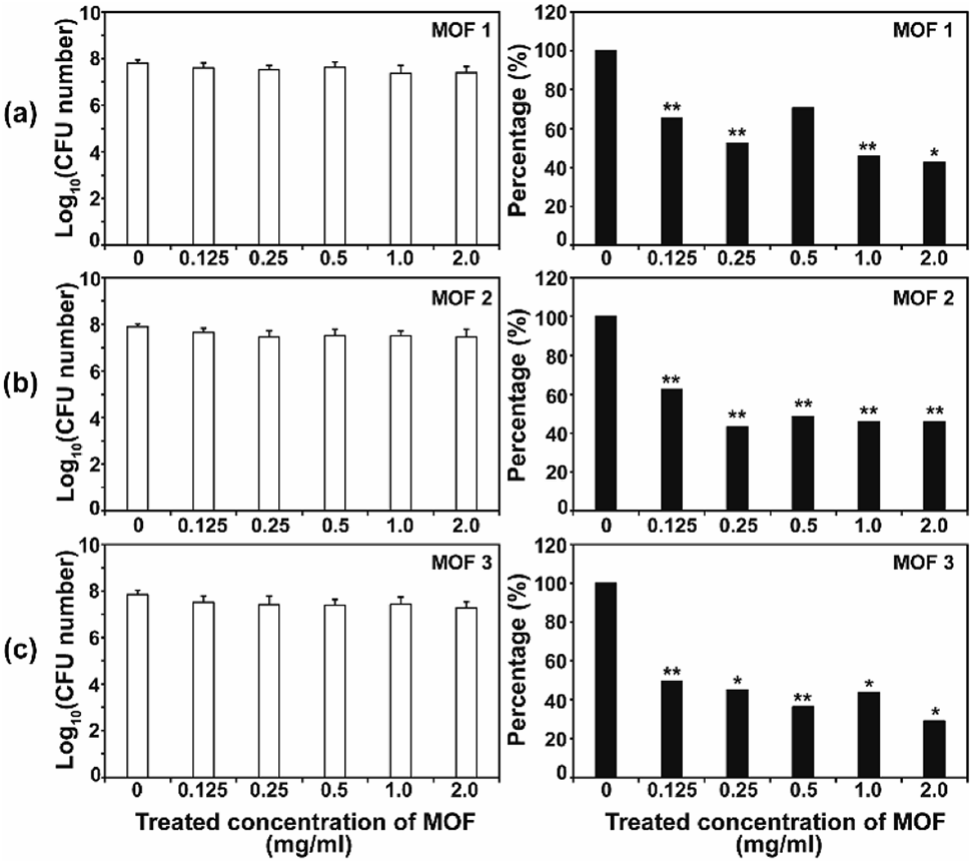
Antifungal activities of MOF 1 (a), MOF 2 (b) and MOF 3 (c) against *C. albicans* at different concentrations. The graphs on the left show the log scale CFU numbers for *C. albicans* in each treatment. The percentages shown in the graphs on the right were calculated as follows; (CFU number in each MOF treatment/CFU number for the control) x 100. Each bar represents the average and standard deviation of nine replicates. A Student's *t*-test was performed for the control and each treatment; *p < 0.05, **p < 0.01.

**Fig. 5:**
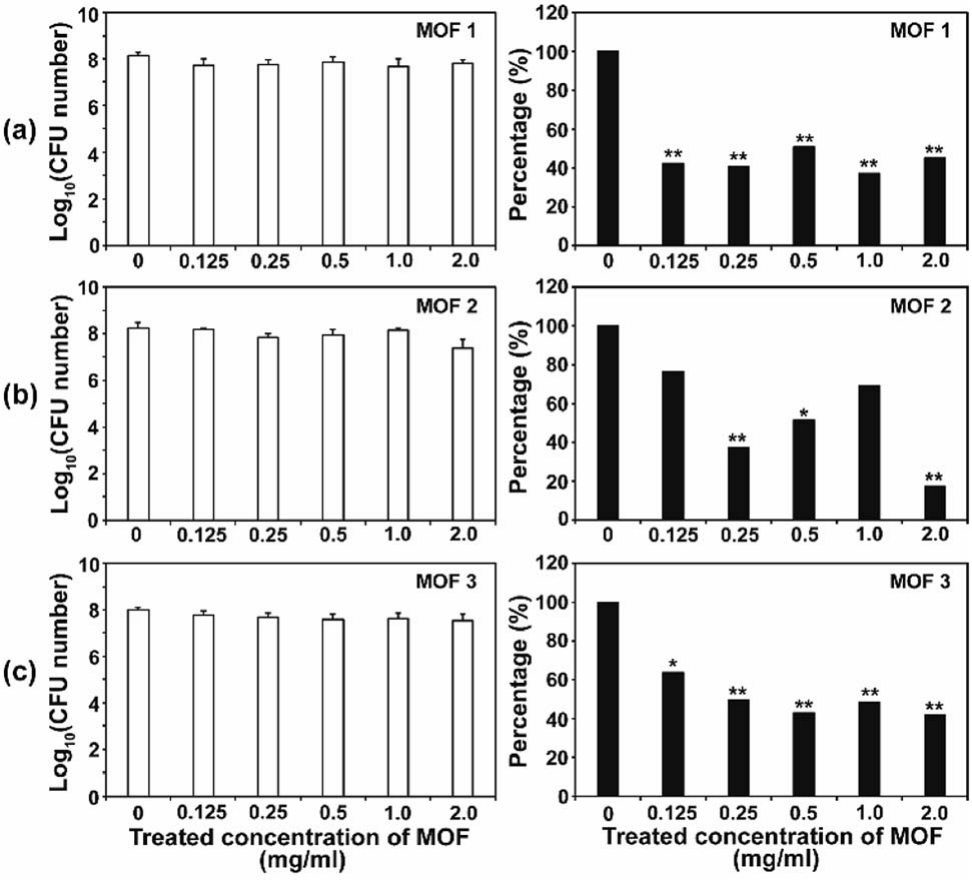
Antifungal activities of MOF 1 (a), MOF 2 (b) and MOF 3 (c) against *A. niger* in different concentrations. The graphs on the left show the log scale CFU numbers for *A. niger* in each treatment. The percentages shown in the graphs on the right were calculated as follows; (CFU number in each MOF treatment/CFU number of control) × 100. Each bar represents the average and standard deviations of nine replicates. A Student's *t*-test was performed for the control and each treatment; *p < 0.05, **p < 0.01.

After the *C. albicans* cells were treated with three types of Cu-MOF for 4 d, the CFU (colony forming unit) number was reduced in a MOF concentration dependent manner (Fig. 4). Approximately 35–50% of the cells were inactivated at 0.125 mg mL^−1^, and a 50–70% reduction was observed at higher concentrations (Fig. 4). Although the efficiency of *C. albicans* inactivation was not significantly different among the different MOFs, 1, 2 and 3, the CFU number was slightly lower after treatment with MOF 3. In *A. niger*, about 60% of the spores were inactivated by 0.125 mg mL^−1^MOF 1, whereas about 25–40% spores were inactivated by 0.125 mg mL^−1^MOF 2 and MOF 3 (Fig. 5). A similar inactivation percentage was maintained in the treatment with 0.25–2.0 mg mL^−1^MOF 1, and a further reduction in spore germination (50–80% reduction) was observed after treatment with 0.25–2.0 mg mL^−1^MOF 2 and MOF 3 (Fig. 5). Upon treatment with MOF 2, the inactivation rate was most significantly decreased, by 80% at a concentration of 2.0 mg mL^−1^ (Fig. 5b). In both *C. albicans* and *A. niger*, no significant difference in the cell inactivation was observed between the samples that were or were not shaken (Fig. S2†).

The antifungal activity of Cu-based MOFs is occasionally reported.^8,9^ Our results provide further evidence to prove that Cu-MOFs can inactivate yeast cells and fungal spores. The efficiency of the fungal inactivation seems to be reduced in our study, compared to that observed by Chiericatti *et al.*^9^ and Bouson *et al.*^8^ in their previously published studies, both found that a higher inactivation rate was achieved with a reduced amount of Cu-MOF in a shorter time. As the structure and properties of the MOFs and the fungal cell numbers used in the assay were different among studies, the fungal inactivation rate can be variable. The inactivation percentage was not significantly different between *C. albicans* and *A. niger*, which showed an average of about 50–70% in this study. This result is different from that found in our previous study using Co-CPs. *C. albicans* showed a greater sensitivity to Co-CPs than *A. niger* under the same experimental conditions (fungal cell number, treatment time, MOF concentration).^11^ A possible explanation for this difference is that overall level of reactive species generated by Cu-MOFs (about 0.05–9 μM for H_2_O_2_, 5–45 μM for NO_*x*_) is lower than that by Co-CPs (0.2–12 μM for H_2_O_2_, 50–500 μM for NO_*x*_).^11^ Although reactive species are not the only factor for antifungal activity of Cu-MOFs, they may have contributed in part to generating the difference in antifungal activity between Co-CPs and Cu-MOFs. Another explanation is that 3D structures of Co-CPs and Cu-MOFs are different, and this may have caused the physical or chemical damage differently in fungal strains.

### 3.3 Apoptosis-like fungal death was observed after treatment with Cu-MOFs

**Fig. 6:**
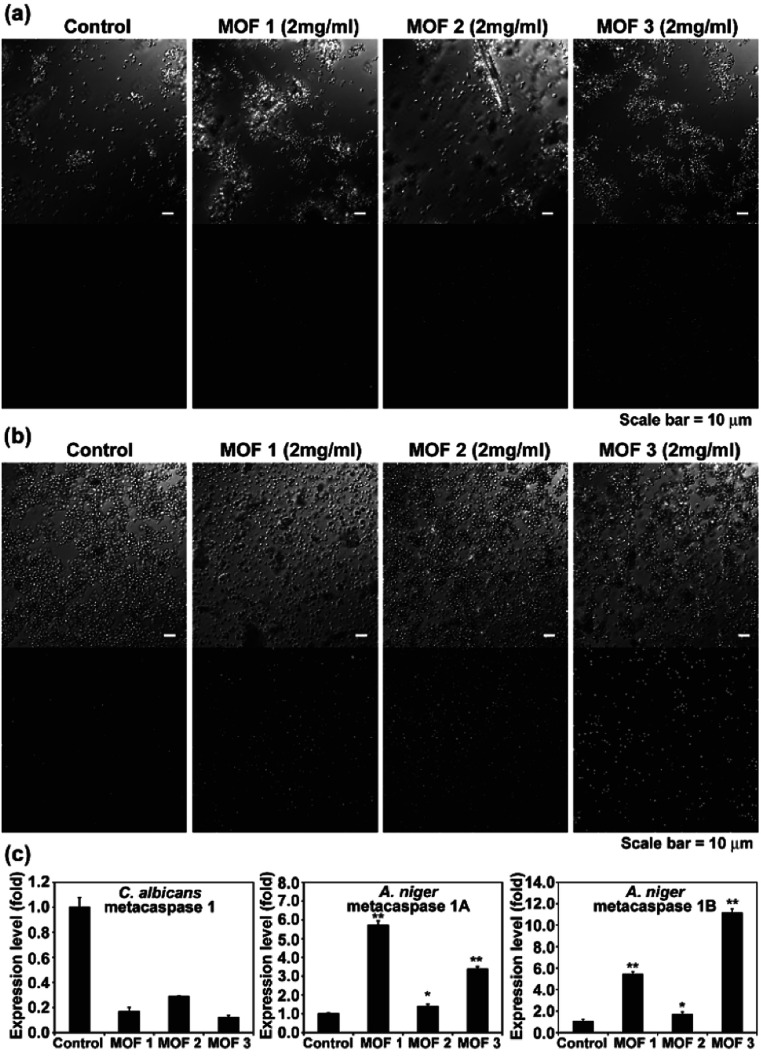
TUNEL assay showing DNA fragmentation (as an indicator of apoptosis-like cell death) in *C. albicans* (a) and *A. niger* (b). (c) Transcription level of the metacaspase gene in *C. albicans* and *A. niger* treated with Cu-MOFs for 1 d. Each value is an average of three replicate measurements. A Student's *t*-test was performed for the control and each treatment; *p < 0.05, **p < 0.01.

We observed apoptosis-like death in *C. albicans* cells and *A. niger* spores after treatment with Cu-MOFs for 4 d (Fig. 6a, b, S3 and S4†). The number of fluorescent (DNA fragmentation) cells and spores were higher in the MOF treated samples compared to the control. The fluorescence was more intense in *A. niger* than *C. albicans* indicating that apoptosis-like death might proceed more readily. In addition, the number of cells and the fluorescence intensity were relatively higher after treatment with MOF 3 compared to MOF 1 and MOF 2 for both *C. albicans* and *A. niger* (Fig. 6a, b, S3 and S4†). We analysed the expression of the metacaspase gene that is known to be activated in fungal apoptosis-like death.^17^ The level of mRNA for metacaspase 1 was much lower than that of the control in the samples treated with Cu-MOFs for 1 d (Fig. 6c). However, the amount of mRNA for metacaspase 1A and 1B was significantly increased in the *A. niger* spores treated with the three types of Cu-MOFs. Treatment with MOF 2 showed the lowest increase in the amount of metacaspase mRNA (Fig. 6c).

Apoptosis-like cell death using MOFs is occasionally observed in human cells, and drug or photodynamic sensitizers encapsulated in MOFs can play a major role in inducing cell apoptosis.^18^ In our results, the apoptosis-like fungal cell death seems to be caused directly by the Cu-MOFs, which is different from the results observed in previous studies. This is the first time that MOFs have been observed to cause apoptosis-like cell death in microorganisms. Our results show that metacaspase transcription is promoted in MOF treated *A. niger*, but not *C. albicans* (Fig. 6c). This may be related to the reduced DNA fragmentation observed in *C. albicans* treated with MOFs in the TUNEL assay (Fig. 6).

### 3.4 Mechanism of the antifungal activity of the Cu-MOFs

**Fig. 7:**
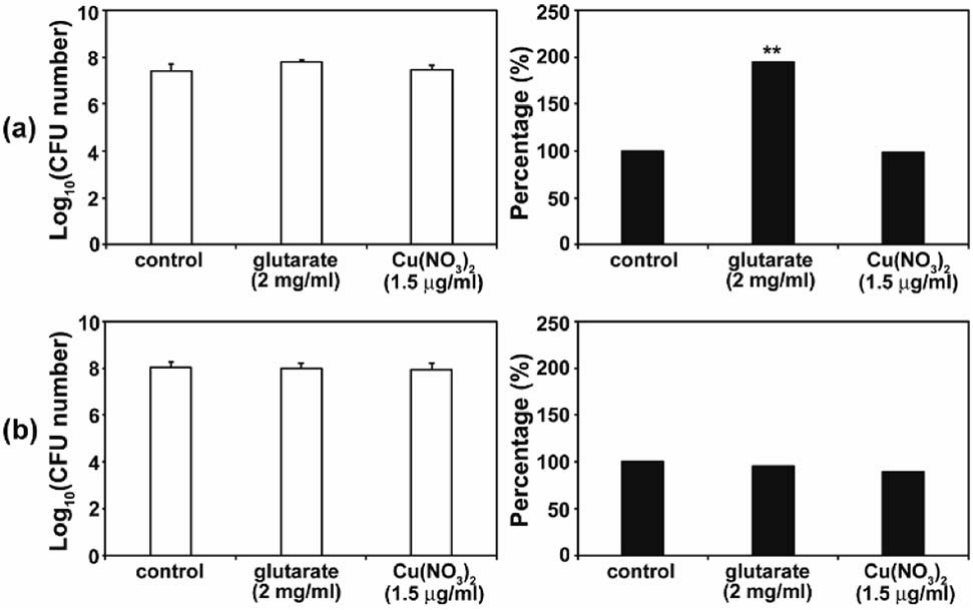
Effect of Cu(NO_3_)_2_ and glutarate on the viability of *C. albicans* (a) and *A. niger* (b). Fungal cells were treated with Cu(NO_3_)_2_ or glutarate for 4 d. Each value represents an average of 18–27 replicates. A Student's *t*-test was performed on the control and each treatment; **p < 0.01.

As the antimicrobial activity of MOFs is known to be attributed to the free metals and organic ligands released from the framework,^19^ we investigated whether free Cu^2+^ and glutarate released from a MOF (Fig. 3) can produce an antifungal effect. Free Cu^2+^ was obtained from the dissolved Cu(NO_3_)_2_ in PBS. When free Cu^2+^ (as Cu(NO_3_)_2_) and glutarate was applied to *C. albicans* cells and *A. niger* spores with the released concentration for 4 d, no significant difference in the fungal CFU number between the control and treatment groups was observed in both types of fungi, except for the treatment of *C. albicans* with glutarate in which the cell number almost doubled after treatment (Fig. 7). This indicates that degeneration of metal and organic ligands is not a critical factor in the fungal inactivation observed after treatment for 4 d. Although free Cu^2+^ itself has antimicrobial effect, the amount released from Cu-MOFs in PBS is not enough to cause inactivation of *C. albicans* cells and *A. niger* spores. Cu-MOFs used in our study is water stable, and therefore, other factors may be more critical than free Cu ion and glutarate. Electric interaction of fungal cells with Cu and glutarate in MOF may possibly cause degeneration of biomolecules on fungal cell surface. Photocatalytic ROS generation from MOF can be another potential mechanism for fungal inactivation.^20^

**Fig. 8:**
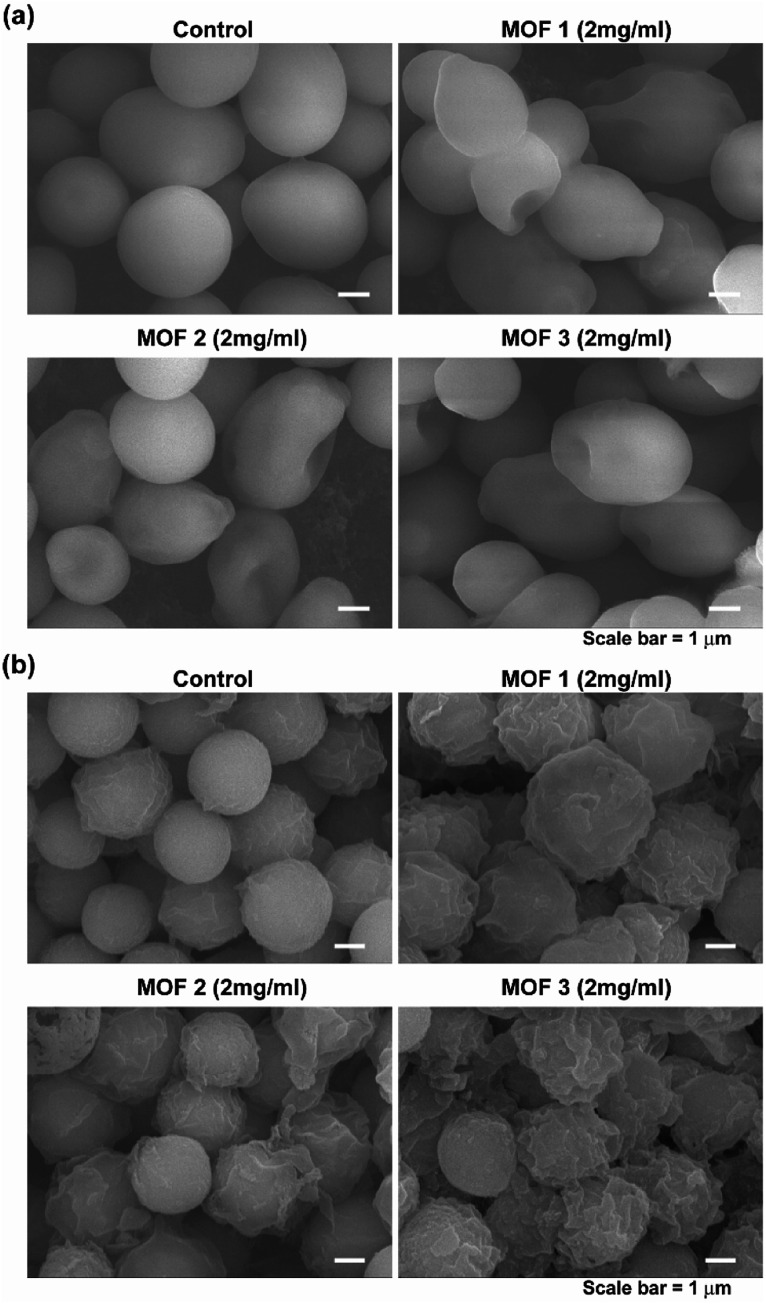
Morphology of the *C. albicans* cells (a) and *A. niger* spores (b) after treatment with MOF 1, MOF 2 and MOF 3 for 4 d.

In the SEM analysis, many *C. albicans* cells treated with the three different types of Cu-MOFs were crushed, compared with the egg shaped control cells (Fig. 8a). No dramatic difference in the cell morphology was observed between the three types of Cu-MOFs. In *A. niger*, most of the spores became crumpled after treatment with the three different types of Cu-MOFs (Fig. 8b). There were barely any crushed cells such as those observed in *C. albicans* (Fig. 8b). The spore surface layer (probably the cell wall) seemed to become rumpled (Fig. 8b).

**Fig. 9:**
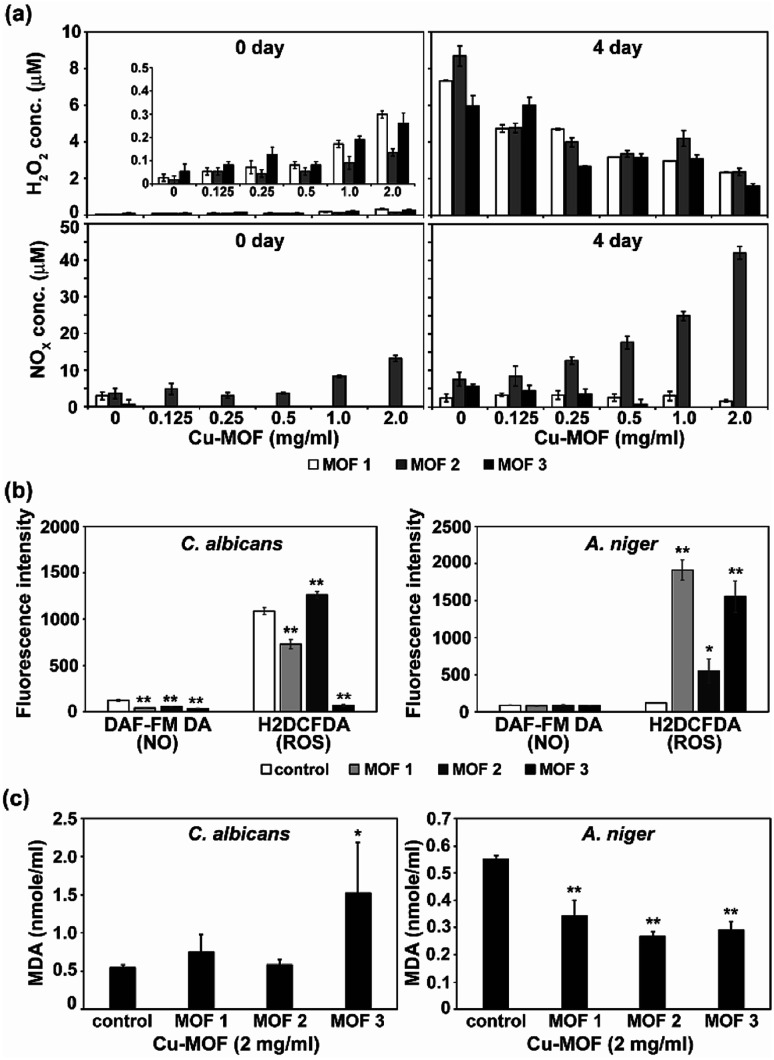
Level of ROS, RNS and lipid peroxidation. (a) Concentration of H_2_O_2_ and NO_*x*_ in PBS 0 and 4 d after treatment with Cu-MOFs. Zoom-in graph of H_2_O_2_ level on 0 d was inserted. (b) Level of intracellular ROS and NO (nitric oxide) in *C. albicans* cells and *A. niger* spores after treatment with Cu-MOFs (2 mg mL^−1^) for 4 d. (c) Peroxidation of the membrane lipid in fungal cells after treatment with the Cu-MOFs for 4 d. Each value represents an average of six replicates. A Student's *t*-test was performed for the control and each treatment; *p < 0.05, **p < 0.01.

MOFs are known to produce ROS, and this ROS can cause an antimicrobial effect.^20^ To test the involvement of reactive species in fungal inactivation, production of ROS and RNS species (H_2_O_2_ and NO_*x*_) in PBS treated with the three different types of Cu-MOFs was examined. Immediately after treatment with the MOFs (0 d), the NO_*x*_ level was found to increase with the increasing concentration of MOF 2 (from about 3 to 13 μM) but this increase was not detected after treatment with MOFs 1 and 3 (Fig. 9a). A relatively low amount of H_2_O_2_ (about 0.05–0.5 μM) was measured and this increased upon treatment with the MOFs in a concentration dependent manner (Fig. 9a). After 4 d, the NO_*x*_ level in the MOF 2 treated PBS was elevated compared to 0 d and increased with upon treatment with the MOFs in a concentration dependent manner (Fig. 9a). The H_2_O_2_ concentration was greater after 4 d compared to 0 d and decreased upon treatment with the MOFs in a concentration dependent manner.

As apoptosis-like death was observed in both *C. albicans* and *A. niger*, the intracellular ROS and RNS level was assessed after treatment with the MOFs. Intracellular NO was not generated after treatment with the MOFs in either fungi (Fig. 9b). However, the level of intracellular ROS detected using H_2_DCFDA was significantly increased in the *A. niger* spores after treatment with all the MOFs, although a relatively lower increase was observed after treatment with MOF 2 (Fig. 9b).

To determine the fungal responses to reactive species produced in PBS, we examined membrane lipid peroxidation. The amount of MDA (the product of membrane lipid peroxidation) was significantly increased in *C. albicans* after treatment with MOF 3 (Fig. 9c). However, it was decreased in *A. niger* after treatment with all of the MOFs (Fig. 9c). This indicates that there was not a dramatic increase in the occurrence of membrane lipid oxidation after treatment with the MOFs.

About the mechanism of the antimicrobial activity of the MOFs, metal ions and organic ligands released from the degraded MOFs are often considered to be critical factors in microbial cell damage and death.^6,19^ Chiericatti *et al.*^9^ demonstrated that Cu ions released from a MOF crystal into the media were responsible for the antifungal effect. In our study, a small amount of Cu ions was released from the frameworks, but the amount was not sufficient to cause fungal inactivation. In addition, glutarate, an organic ligand chemical used in our study, was not found to be harmful to fungal cells and spores at the concentrations used in the construction of the MOF.

Several studies have demonstrated that the reactive species generated by MOF-mediated photooxidation are responsible for antimicrobial activity.^11,19,20^ In our results, the H_2_O_2_ level was increased immediately after treatment with Cu-MOFs in a concentration dependent manner, however, the level was lower than that in the control after 4 d. H_2_O_2_ produced immediately after treatment with MOFs may play a role in fungal inactivation. However, it may not be sufficient to cause fungal inactivation after 4 d. The level of NO_*x*_ in PBS was only found to be increased in MOF 2, and therefore, cannot be considered as a common factor generating an antifungal effect. In addition, membrane lipid peroxidation does not seem to significantly occur in treated fungal cells. These results suggest that the reactive species generated in PBS by Cu-MOFs may not be critical to fungal inactivation. In addition, generation of H_2_O_2_ and NO_*x*_ by Cu-MOFs was much lower than that by Co-MOFs.^11^ This may explain the high sensitivity of *C. albicans* to Co-MOFs than Cu-MOFs.^11^

We observed a significant increase in the level of intracellular ROS in *A. niger* spores. This may be related to the slightly higher level of apoptosis-like death observed in *A. niger* compared to *C. albicans*. In addition, transcription of the metacaspase gene was significantly increased in *A. niger* treated with MOFs. Cu-MOF treatment may have caused the elevation of the intracellular ROS level in *A. niger*, and this could promote metacaspase gene expression, leading to apoptosis-like cell death. However, the higher rate of apoptosis-like death in the MOF 3 treated cells still cannot be explained. Further investigation on this is needed.

## 4. Conclusions

Three types of 3D Cu-MOFs containing glutarates and bipyridyl ligands (bpa, bpe, or bpp) were synthesized by using previously reported hydrothermal reactions or a layering method. All three Cu-MOFs contained well-defined 1D channels with very similar pore shapes and different pore dimensions. The bulk purities of the Cu-MOFs were confirmed using PXRD and IR spectra. The antifungal activity of the Cu-MOFs against *C. albicans* cells and *A. niger* spores showed a similar efficiency. However, our study also demonstrated that the molecular mechanisms for fungal inactivation of the Cu-MOFs were slightly distinct between the different fungal species. Although an average of about 50–80% fungal inactivation by Cu-MOFs was observed in our experimental conditions, our results suggest that Cu-MOFs have potential for application as antifungal agents that could be used in various contamination or infection situations.

## Conflicts of interest

There are no conflicts to declare.

## Supplementary Material

RA-011-D0RA08743B-s001
