# Supplementary material for: Analysis of the effects of Cu-MOFs on fungal cell inactivation
Source: RSC Adv. 2021 Jan 4;11(2):1057–65. doi: 10.1039/d0ra08743b (PMC8693517; doi:10.1039/d0ra08743b)
Supplement: RA-011-D0RA08743B-s001 [file RA-011-D0RA08743B-s001.pdf]

Electronic Supplementary Material (ESI) for RSC Advances.
This journal is © The Royal Society of Chemistry 2020

Supplementary Information

Analysis for the effects of Cu-MOFs on fungal cell inactivation

Mayura Veerana,†[a] Hyun-Chul Kim,†[b] Sarmistha Mitra,[a] Bishwa Chandra Adhikari,[a] Gyungsoon Park,[a] Seong Huh,[b] Sung-Jin Kim,[c] and Youngmee Kim*[c]*

[a] *Plasma Bioscience Research Center and Department of Electrical and Biological Physics, Kwangwoon University, Seoul 01897, Republic of Korea. E-mail: gyungp@kw.ac.kr*

[b] *Department of Chemistry and Protein Research Centre for Bio-Industry, Hankuk University of Foreign Studies, Yongin 17035, Korea*

[c] *Institute of Nano-Bio Technology and Department of Chemistry and Nano Science, Ewha Womans University, Seoul 120-750, Korea. E-mail: ymeekim@ewha.ac.kr; Fax: +82-2-3277-2385; Tel: +82-2-3277-4164*

† *These authors contributed equally to this work.*

Correspondence and requests for materials should be addressed to Y.K. (email: ymeekim@ewha.ac.kr) or to G.P. (email: gyungp@kw.ac.kr).

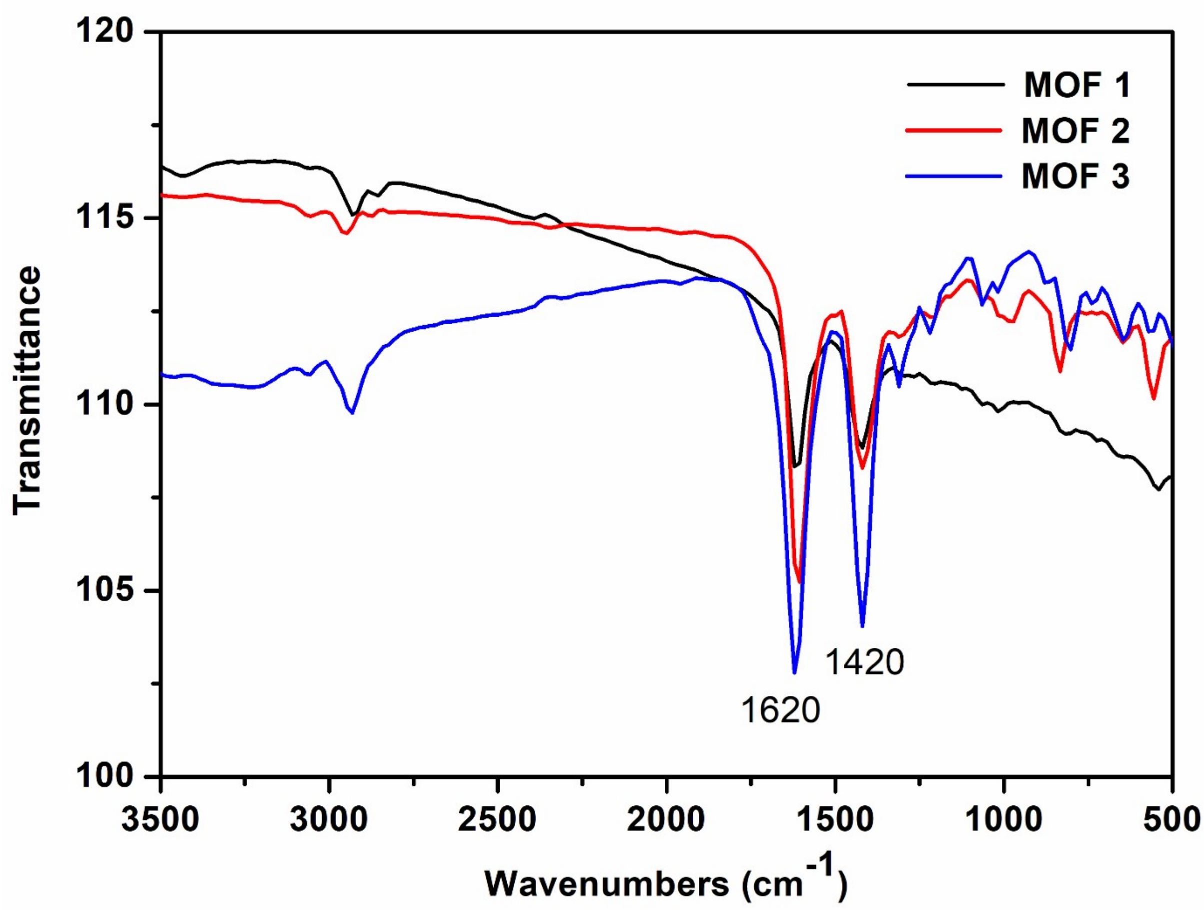

Fig. S1. IR spectra of of Cu-MOFs **1** - **3**.

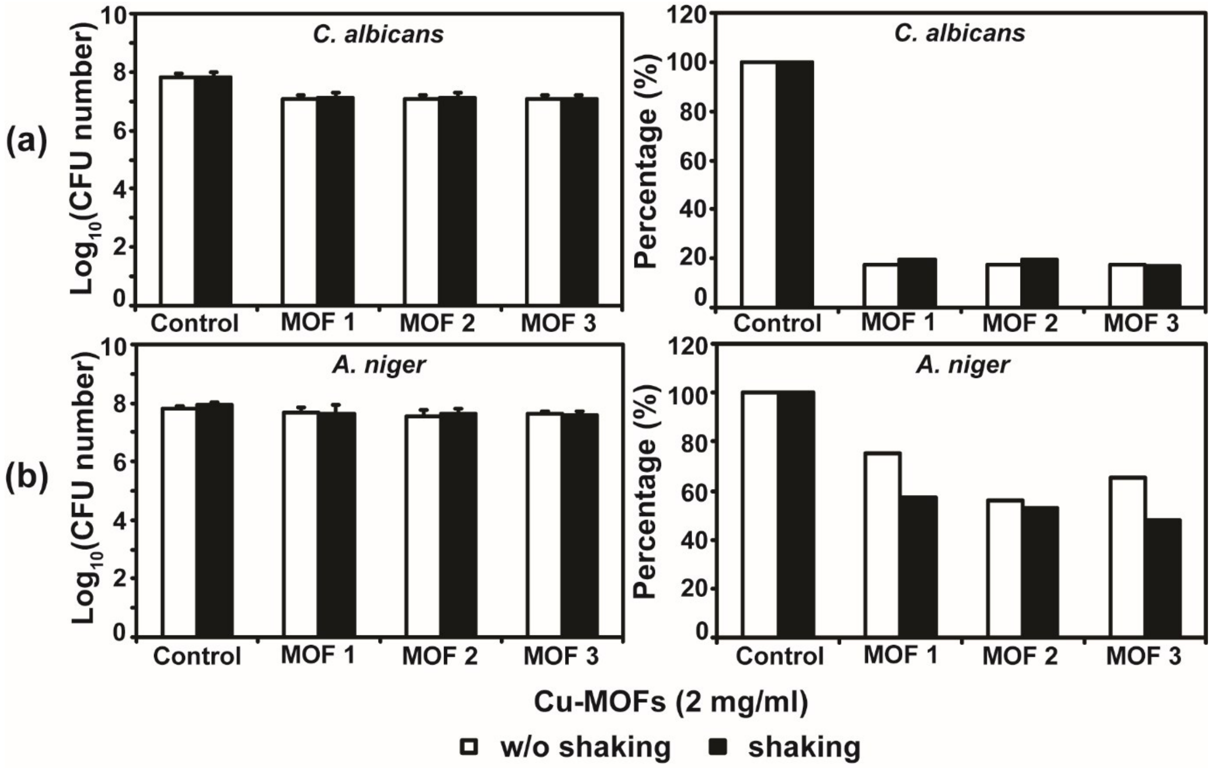

Fig. S2. Effect of collision after shaking Cu-MOFs on viability of *C. albicans* (a) and *A. niger* (b) cells at 4 days.

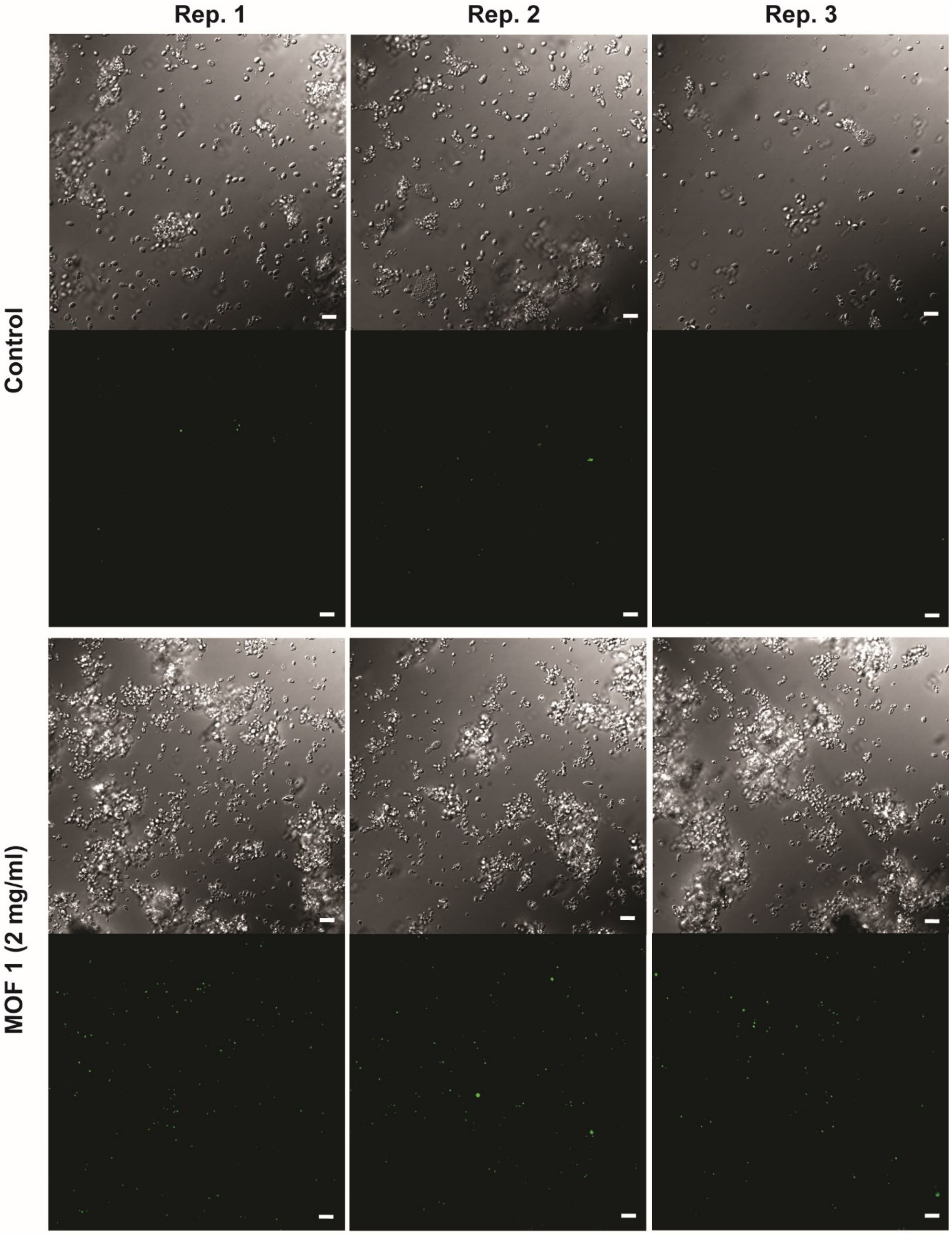
Rep. 1
Rep. 2
Rep. 3
Control
MOF 1 (2 mg/ml)
Scale bar = 10 µm

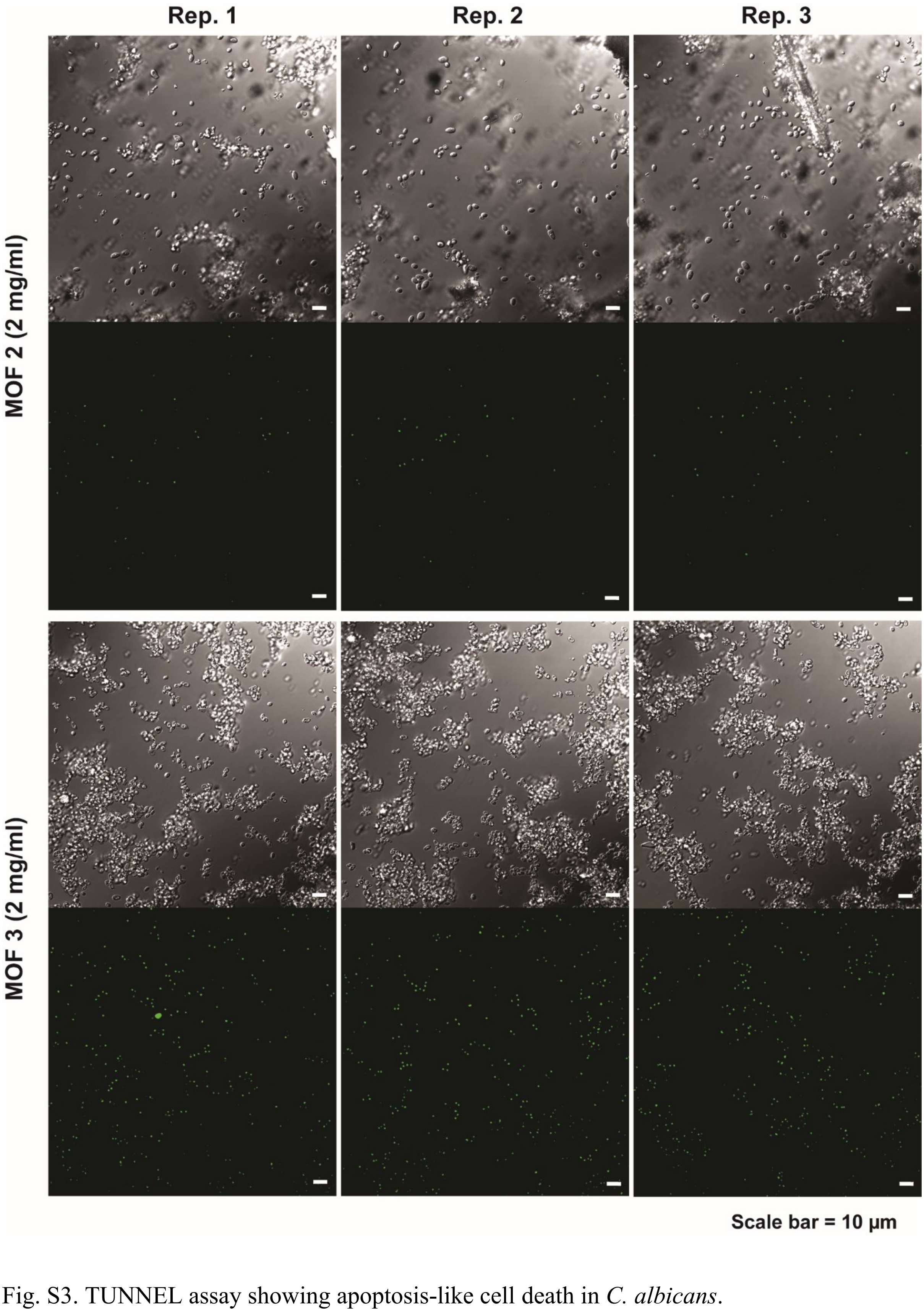

Fig. S3. TUNNEL assay showing apoptosis-like cell death in *C. albicans*.

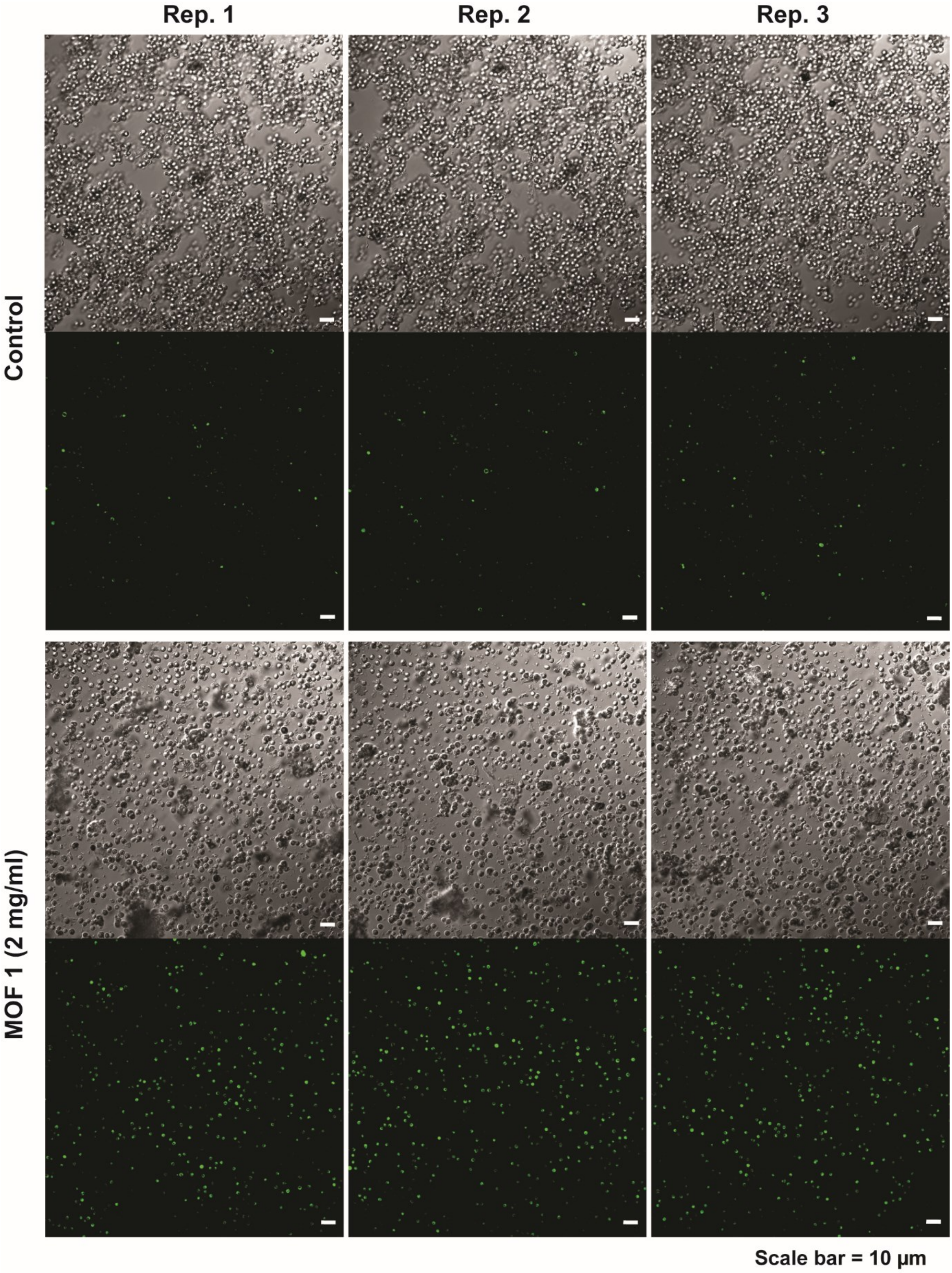
Rep. 1
Rep. 2
Rep. 3
Control
MOF 1 (2 mg/ml)
Scale bar = 10 μm

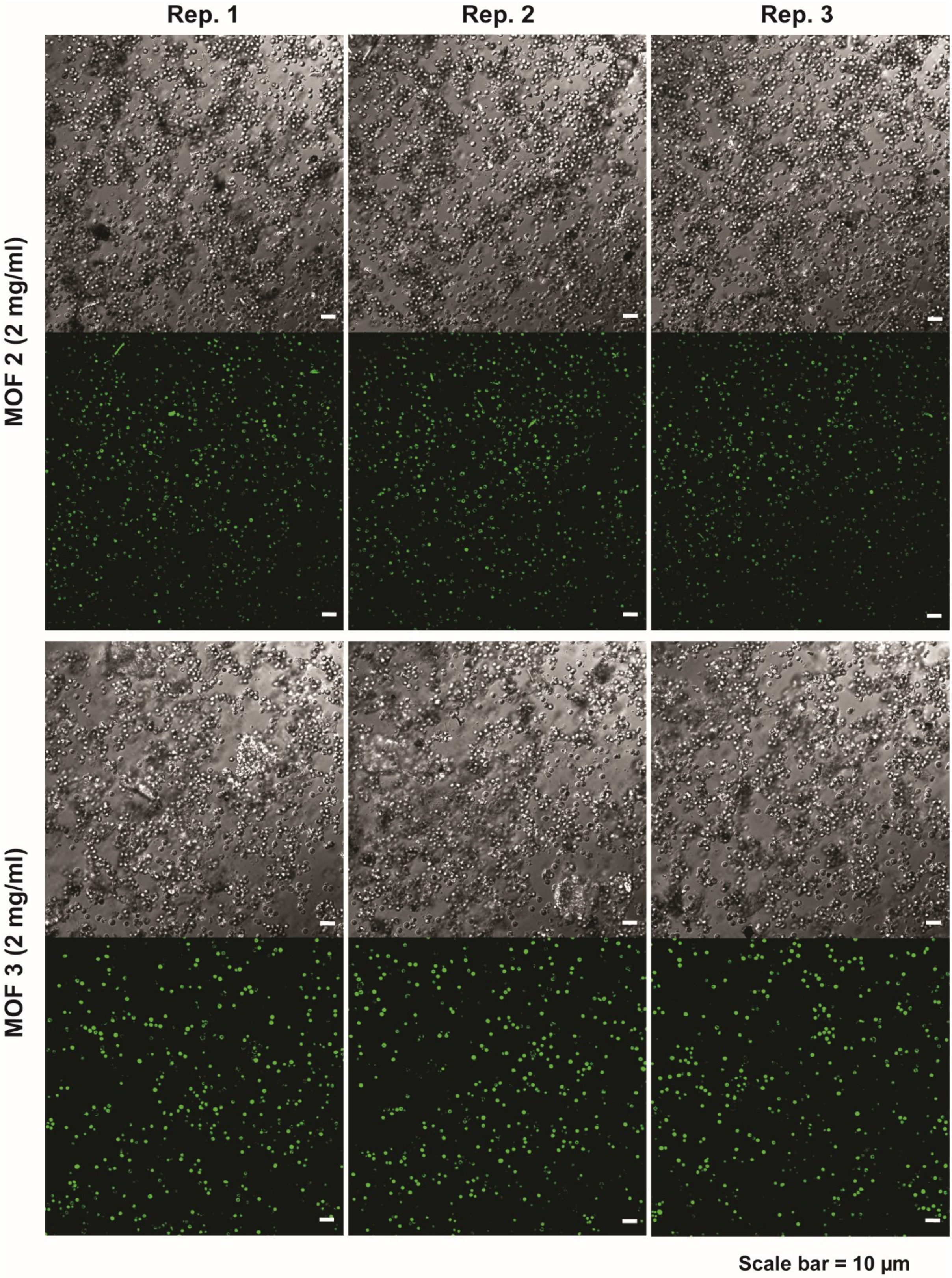

Fig. S4. TUNNEL assay showing apoptosis-like cell death in *A. niger*.
